# Automated three-dimensional activation versus conventional mapping for catheter ablation of atrial tachycardia – A prospective randomized trial

**DOI:** 10.1016/j.ijcha.2023.101222

**Published:** 2023-05-24

**Authors:** Raphael Spittler, Niclas Witte, Boris Alexander Hoffmann, Alexandra Marx, Hanke Mollnau, Blanca Quesada-Ocete, Torsten Konrad, Thomas Rostock

**Affiliations:** Center of Cardiology, Department of Cardiology II – Electrophysiology, University Medical Center Mainz, Mainz, Germany

**Keywords:** Atrial tachycardia, Ablation, Electroanatomic map, Arrhythmia recurrence

## Abstract

**Background:**

The automated NavX Ensite Precision latency-map (LM) algorithm aims to identify atrial tachycardia (AT) mechanisms. However, data on a direct comparison of this algorithm with conventional mapping are scarce.

**Methods:**

Patients scheduled for AT ablation were randomized to mapping with the LM- algorithm (LM group) or to conventional mapping (conventional only group: ConvO), using entrainment and local activation mapping techniques. Several outcomes were exploratively analyzed. Primary endpoint was intraprocedural AT Termination. If AT termination with only automated 3D-Mapping failed, additional conventional methods were applied (conversion).

**Results:**

A total of 63 patients (mean 67 years, 34 % female) were enrolled. In the LM group (n = 31), the correct AT mechanism was identified in 14 patients (45 %) using the algorithm alone compared to 30 patients (94 %) with conventional methods. Time to termination of the first AT was not different between groups (LM group 34 ± 20 vs. ConvO 43.1 ± 28.3 min; p = 0.2). However, when AT termination did not occur with LM algorithm, time to termination prolonged significantly (65 ± 35 min; p = 0.01). After applying conventional methods (conversion), procedural termination rates did not differ between LM group (90 %) vs. ConvO (94 %) (p = 0.3). During a follow-up time of 20 ± 9 months, no differences were observed in clinical outcomes.

**Conclusion:**

In this small prospective, randomized study, the use of the LM algorithm alone may lead to AT termination, but less accurate than conventional methods.

## 1 Introduction

Atrial tachycardias (AT) are increasingly occurring, particularly after complex atrial fibrillation ablations [1]. Three-dimensional electroanatomic mapping (EAM) is widely used in electrophysiological procedures aiming at catheter ablation of complex arrhythmia. The application of EAM is considered to reduce procedure duration and radiation exposure [2]. However, as procedure complexity increases, this benefit has been increasingly debated with the argument that it seems to disappear in more complex ablations, such as AT ablation procedures [3], [4].

Current EAM systems can visualize propagation of AT with specific automated algorithms. These mapping algorithms aim to identify the AT mechanism and thereby provide the anatomical location of the AT area critical for tachycardia maintenance [5]. This prospective randomized trial sought to compare conventional electrophysiological techniques using activation and entrainment mapping with an automated algorithm-based activation mapping approach (NavX Ensite Precision Cardiac Mapping System, Abbott Medical, Chicago, IL, USA).

## 2 Methods

### 2.1 Study population

Between March 2016 and June 2019, a total of 67 patients were assessed for study inclusion at a single tertiary care center (University Medical Center Mainz, Germany). Inclusion criteria were de-novo AT or AT after complex persistent atrial fibrillation (persAF) ablation, and ongoing AT immediately prior the procedure. Exclusion criteria were termination or degeneration in AF before AT mapping. All antiarrhythmic drugs, except for amiodarone, were discontinued at least 5 half-lifes prior to the procedure. The study was approved by the local ethics committee and complied with the provisions of the Declaration of Helsinki. All patients provided written informed consent.

### 2.2 Randomization protocol

Patient were assessed for eligibility prior to ablation. If patients were in AT immediately prior to the procedure, they were randomized (1:1) to latency-map (LM) group or conventional only group (ConvO). A 3-D electroanatomical map with the EnSite NavX system was created in all patients, irrespective of randomization. In patients randomized to the ConvO, only an anatomical map shell was displayed without any activation mapping information, mapping of AT was performed by using conventional electrophysiological mapping techniques exclusively. In the LM group, the information provided by the automated activation mapping was analyzed to define the ablation strategy.

**Fig. 1:**
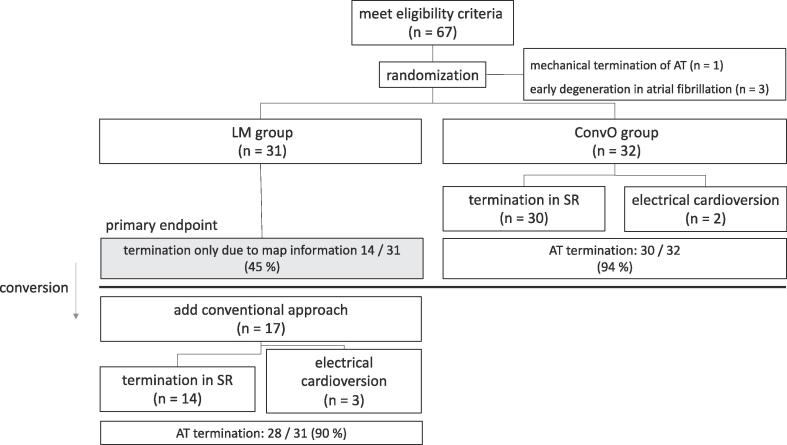
Study flow chart. Conversion involves the use of conventional methods such as entrainment mapping or manual measurement of local activation time if the termination of the AT fails due to the LM algorithm alone. SR: sinus rhythm. AT: atrial tachycardia. EAM: Electroanatomic map. LM: latency-map.

If ablation at the critical area of AT as defined by the LM-algorithm did not result in AT termination, the primary endpoint of procedural AT termination was not achieved. Additional conventional mapping techniques were then applied (conversion) to identify the correct AT mechanism and the critical site for AT termination. The study flowchart is presented in Fig. 1.

### 2.3 Electrophysiological procedure and catheter ablation

All patients underwent a standardized procedure under sedation using propofol and fentanyl as per local standard of care. All patients received transesophageal echocardiography prior to the procedure to exclude atrial thrombi. Details of the procedural approach have been described previously [6]. In brief, the following catheters were introduced via a femoral vein access: (1) A steerable decapolar catheter (Inquiry^TM^, IBI, Irvine Biomedical, Inc., Irvine, CA, USA) was positioned within the coronary sinus (CS); (2) a circumferential decapolar diagnostic catheter (Lasso, Biosense-Webster, Diamond Bar, CA, USA) for mapping; and (3) a 3.5 mm externally irrigated-tip ablation catheter (Biosense-Webster, Diamond Bar, CA, USA) was used for mapping and ablation. A single bolus of 100 IU/kg body weight heparin was administered after transseptal puncture. The activated clotting time (ACT) was assessed every 30 min and maintained within 250–350 s.

In all patients, an EAM was created with the Lasso catheter (10 pole) using the EnSite NavX system. The first step of the ablation procedure, irrespective to the randomization result, was mapping and re-isolation of the pulmonary veins (PVs), if a pulmonary vein isolation (PVI) had been performed previously. After PV re-isolation, the Lasso catheter was placed in the left atrial (LA) appendage (LAA).

In the LM group, the parameters of the automated activation mapping software were set as follows: the window-of-interest was set with a total width of 90 % of the AT cycle length and with half of the window preceding P-wave onset and the other half following P-wave onset. In case of AT without having clearly separated P-waves, the earliest CS activation was used as a reference for determining interval boundaries. A detailed evaluation of the mapping results gathered by the automated software algorithm was performed using a propagation map.

In patients randomized to the ConvO, only conventional electrophysiological criteria were used to evaluate the AT mechanism. For conventional mapping, we used the principles of the deductive mapping strategy as described previously [7]. In brief, the first step was the evaluation of cycle length regularity. When the AT had a regular cycle length, a macro-reentrant mechanism was evaluated by using local activation sequences of the coronary sinus, LAA and the ablation catheter sequentially placed at the anterior, posterior, and lateral LA, respectively. Entrainment mapping was performed from the CS, the LAA and at the above-described areas with the ablation catheter. When a focal AT mechanism was assumed, a detailed conventional activation mapping targeting the earliest endocardial activation in relation to a fixed intracardiac electrogram, most commonly a coronary sinus electrogram, was performed. Furthermore, the local activation as compared to P-wave onset was analyzed.

Catheter ablation was performed uniformly for the identified AT mechanism, irrespective of the randomization group. For macro-reentrant AT, linear ablation was performed with the aim of connecting two anatomical or electrophysiological boundaries. Focal AT was targeted with discrete ablation at the site of earliest endocardial activation, identified by either conventional mapping or the LM algorithm.

The primary study endpoint was the rate of AT termination. Additional secondary endpoints included total procedure time, fluoroscopy time, and time to AT termination. Total procedure time was defined as the duration from vascular groin access to sheath removal. Time to AT termination was defined as the interval from the initiation of the respective mapping technique to the termination of AT.

The procedural endpoint was termination of the clinical AT and elimination of all consecutively inducible ATs. All procedures were performed only by experienced operators (>1000 performed procedures).

### 2.4 Follow up

All patients were seen in our outpatient department at regular intervals with 48-h or 72-h Holter ECGs. In addition, the patients received 24 h Holter ECGs as well as 12-lead ECGs by their referring physicians. This was supplemented by telephone follow-up interviews. In case of symptoms suggestive of arrhythmia recurrences, additional visits were performed at our institution. Any documented arrhythmia in Holter ECGs (lasting > 30 s) or 12-lead ECG was considered arrhythmia recurrence. A blanking period of 3 months was used.

### 2.5 Statistical analysis

Continuous variables are specified as mean with standard deviation or median and interquartile range (IQR) as appropriate. Statistical significance was estimated with Students-*t*-test or Mann-Whitney *U* test if normal distribution was rejected. Test for normal distribution was performed using Shapiro-Wilk test. Categorical variables were analyzed with the Fisher exact test, and post-hoc analysis for multiple hypotheses testing was performed with the Benjamini-Hochberg FDR method. Comparison of categorical variables was performed with odds-ratios and by indicating confidence intervals. Time dependent arrhythmia recurrence was investigated with Kaplan-Meier analysis and log-rank-test. All significance tests were two-tailed with rejecting the null-hypothesis at p < 0.05. Statistical analysis was performed with R (R Foundation for Statistical Computing, R Development Core Team, Vienna, Austria).

## 3 Results

### 3.1 Patient characteristics

A total of 67 patients with AT were enrolled for eligibility. Four patients were excluded from the analysis because of mechanical termination of AT during mapping in one patients and conversion to AF during mapping in another three patients. Therefore, the primary analysis included 63 patients (31 patients in the LM group, and 32 patients of the ConvO) (Table 1). The mean patient age was 67 ± 10 years, 34 % were female, and the mean body mass index (BMI) was 27.9 ± 3.9 kg/m^2^. A total of 93 % of patients had at least one pre-procedure.

**Table 1:** Baseline characteristics.

Clinical parameter	ConvO (n = 32)	LM group (n = 31)	Overall (n = 65)	p
age, years	69 ± 8	65 ± 11	67 ± 10	0.1
female, n (%)	14 (44)	7 (23)	23 (35)	0.1
BMI, kg/m^2^	28.3 ± 4.2	27.4 ± 3.6	27.9 ± 4.0	0.6
Number of preprocedures	1.4 ± 0.6	1.8 ± 1.2	1.6 ± 1.0	0.2
Number of ecv (overall), n	3.8 ± 2.6	3.4 ± 3.4	3.7 ± 3.0	0.5
CHA_2_DS_2_-VASc-Score	3.2 ± 1.8	3.0 ± 1.7	3.1 ± 1.8	0.8
AAD, n (%)	9 (28)	7 (22)	17 (23.9)	0.5
DOAC, n (%)	24 (75)	19 (57)	43 (66.1)	0.1
Phenprocoumon, n (%)	8 (25)	14 (42)	22 (33.9)	0.1
AT duration, months	2.4 ± 2.4	1.3 ± 1.7	1.8 ± 2.1	0.1
Echocardiographic parameter				
Body-surface-area, m^2^	2.3 ± 0.3	2.4 ± 0.3	2.3 ± 0.3	0.1
IVSd, cm	1.2 ± 0.2	1.3 ± 0.3	1.2 ± 0.2	0.1
LVIDd, cm	4.4 ± 0.7	4.6 ± 0.7	4.5 ± 0.7	0.4
PWT, cm	1.3 ± 0.3	1.4 ± 0.5	1.3 ± 0.4	0.5
LV-mass-index, g/m^2^	90 ± 23	100 ± 36	95 ± 30	0.5
LA-diameter, cm	4.3 ± 0.6	4.5 ± 0.7	4.4 ± 0.7	0.5
LA-volume-index, ml/m^2^	30 ± 16	31 ± 14	31 ± 15	0.7
Mitral regurgitation, n (%)	19 (59.4)	16 (48.5)	34 (54)	0.5
LAA-flow, cm/s	42 ± 16	53 ± 19	47 ± 18	0.01
Ejection fraction, (%)	51 ± 11	46 ± 12	49 ± 12	0.1

LM = latency-map; BMI = body mass index; AAD = antiarrhythmic drugs; DOAC = direct oral anticoagulant; AT = atrial tachycardia; IVSd = intraventricular septum thickness end diastole; LVIDd = left ventricular internal diameter end diastole; PWT = posterior wall thickness; LV = left ventricle; LAA = left atrial appendage. ConvO = conventional only group.

### 3.2 Main findings

In the LM arm, the mean mapping time was 8 ± 4 min. The median (IQR) number of EAM points acquired was 555 (227–1119) of which 374 (180–691) points were used for AT mechanism analysis. In 18 patients (58 %), the EnSite NavX system identified a specific AT mechanism.

The primary endpoint (termination of the first AT) was achieved in 14 patients (45 %) using LM only, and in 30 patients (94 %) using conventional methods (P = 0.03) (Table 3). In the LM group, the rate of first AT termination increased to 90 % with the additional use of conventional methods (Fig. 1). AT termination was not achieved in 5 patients (3 in the LM group, 2 in the ConvO). Of those, 3 patients converted to AF during ablation and 2 patients had multiple conversions between the clinical and a different AT during ablation, preventing accurate evaluation of the AT mechanism.

At the end of the procedure, the mean number of ATs ablated was 1.9 ± 1.0 in LM group, and 1.3 ± 0.5 in the conventional group (p < 0.01). The overall mean procedure time was significantly longer in the LM group than in the conventional group (120 ± 55 vs. 93 ± 38 min; p = 0.03). In patients in whom AT termination was achieved using the LM alone, the time to termination of the first AT was 34 ± 20 min vs. 43 ± 28 min using conventional methods (p = 0.14).

**Fig. 2:**
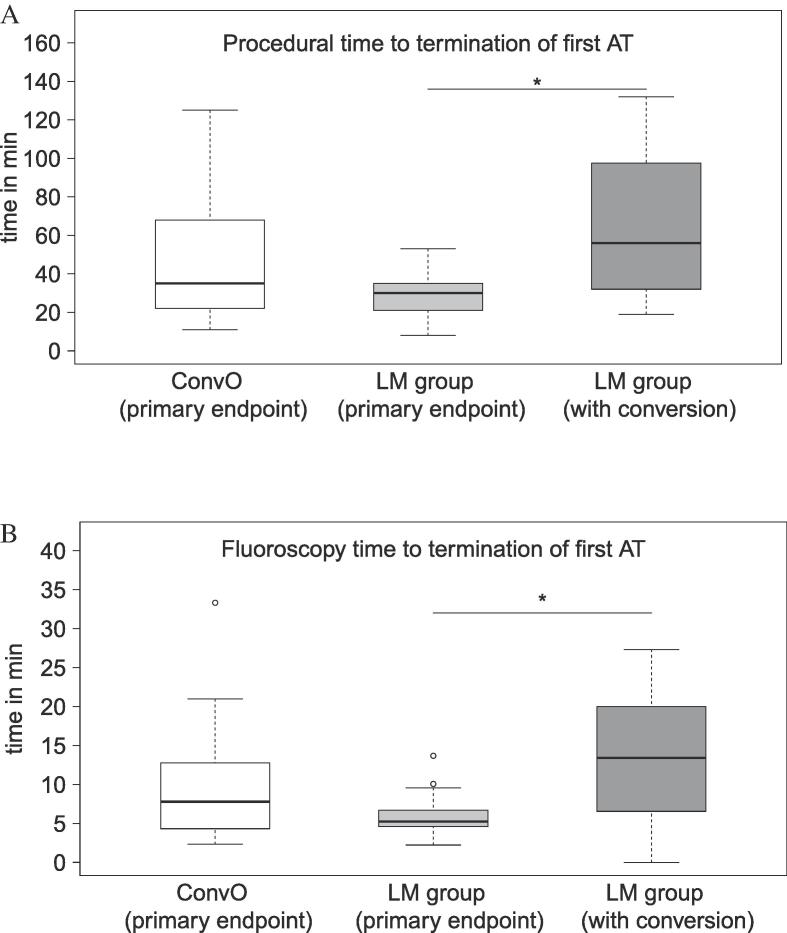
Comparison of the primary endpoint (termination of the first AT) between the two study groups (ConvO and LM group) in terms of time to termination (panel A) and fluoroscopy time (panel B). No statistical difference was found. However, the comparison of patients of the LM group who required additional conventional mapping to achieve AT termination (LM group with conversion) with EAM patients achieving the primary endpoint, time to termination and fluoroscopy time was significantly different (*p = 0.01). Furthermore, the variability of standard deviation of time to termination and fluoroscopy time was significantly lower in the LM group as compared to the ConvO (p = 0.003). LM: latency-map. AT: atrial tachycardia. EAM: Electroanatomic map. ConvO: conventional methods only group.

The mean fluoroscopy time in the LM group was significantly longer than in conventional group (20 ± 13 vs. 12 ± 9 min, p = 0.001). The increase in procedure and fluoroscopy time in the LM group was attributed to the subgroup in which additional conventional methods were used. Fig. 2.

### 3.3 Mechanisms of atrial tachycardia

**Fig. 3:**
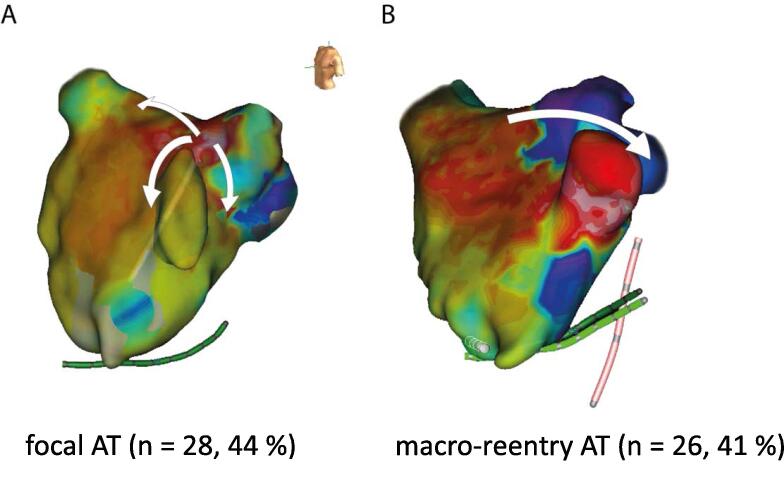
Examples of typical map patterns created with the latency algorithm. Panel A shows a LAO view of the left atrium (LA). The activation map reveals a focal atrial tachycardia (AT) arising from the anterior part of the left superior pulmonary vein. Panel B is an example of the “early-meets-late” pattern from a macro-reentry. The isthmus was identified at the LAA ridge, where successful AT termination was achieved. LAO: left anterior oblique; LAA: left atrial appendage, AT: atrial tachycardia.

The maps revealed the typical electroanatomical activation patterns of consecutive ATs after AF ablation as described in previous studies with the majority having a focal origin[8] (Fig. 3).

The most common types of AT mechanisms in the overall study population were focal AT (n = 28, 44 %) and macro-reentrant AT (n = 26, 41 %). (Fig. 3) Localized reentrant AT was observed in only 9 (15 %) patients. There was no difference in the distribution of AT types between the two groups (Table 2). Of the 26 macro-reentrant ATs, 8 (31 %) were common type right atrial flutter, 8 (31 %) mitral-isthmus-dependent AT, 5 (19 %) LA roof-dependent AT and 5 (19 %) AT had a left atrial anterior circuit.

**Table 2:** Electrophysiological findings.

	ConvO (n = 32)	LM group (n = 31)	Total (n = 63)	p	OR	95 %-CI
Type of AT						
Focal, n (%)	14 (44)	14 (45)	28 (44)	0.9	1.0	0.3 – 3.0
Macro-reentry, n (%)	14 (44)	12 (39)	26 (41)	0.9	0.8	0.2 – 2.7
Localized-reentry, n (%)	4 (12)	5 (16)	9 (15)	0.9	1.3	0.2 – 7.2
Number of treated ATs	1.3 ± 0.5	1.9 ± 1.0	1.62 ± 0.86	< 0.01	–	–
Mitral-Isthmus-line						
First-time block, n (%)	10 (31)	9 (29)	19 (30)	0.9	0.9	0.3 – 3.0
Re-block, n (%)	3 (9)	6 (19)	9 (14)	0.3	2.3	0.4 – 15.6
Persistent block, n (%)	9 (28.1)	5 (16)	14 (22)	0.3	0.5	0.1 – 1.9
Roof-Line						
First-time block, n (%)	6 (19)	5 (16)	11 (17)	0.9	0.8	0.2 – 3.8
Re-block, n (%)	3 (9)	1 (3)	4 (6)	0.6	0.3	0.01 – 4.3
Persistent block, n (%)	9 (28)	10 (32)	19 (30)	0.8	1.2	0.4 – 4.1
CTI						
First-time block, n (%)	2 (6)	6 (19)	8 (13)	0.1	3.5	0.6 – 38.7
Re-block, n (%)	5 (16)	6 (19)	11 (17)	0.8	1.2	0.3 – 6.1
Persistent block, n (%)	7 (22)	4 (13)	11 (17)	0.5	0.5	0.1 – 2.4
Re-PVI, n (%)	10 (31)	6 (19)	16 (25)	0.4	0.5	0.1 – 1.9

CTI = cavotricuspid isthmus; PVI = pulmonary vein isolation; OR = odds ratio; CI = confidence interval, all other abbreviations see Table 1.

**Table 3:** Endpoints.

	ConvO (n = 32)	LM group (n = 31)	Total (n = 63)	p	OR	95 %-CI
Total procedure time, min	93 ± 38	120 ± 55	106 ± 49	0.03	–	–
Time to termination of first AT, min	46 ± 30	53 ± 34	49 ± 33	0.4	–	–
Fluoroscopy time to termination of first AT, min	10 ± 7	11 ± 8	10 ± 7	0.5	–	–
Termination in sinus rhythm, n (%)	30 (94)	28 (90)	92	0.7	0.6	0.05 – 6.30
Total fluoroscopy time, min	12 ± 9	20 ± 14	16 ± 12	< 0.01	–	–
Radiation dose, cGycm^2^	409 ± 283	611 ± 486	508 ± 406	0.05	–	–
Pericardial tamponade, n (%)	1 (3.1)	0	–	–	–	–
AV-fistula (groin), n (%)	1 (3.1)	0	–	–	–	–
Follow-up time, months	19 ± 9	22 ± 9	20 ± 9	0.09	–	–
Re-Procedures after AT-Ablation, n	0.4 ± 0.6	0.5 ± 0.9	0.5 ± 0.7	0.6	–	–

AV: Atrio-venous, all other abbreviations see Table 1.

Procedural termination in the LM group based on latency algorithm information was possible in 9 of 15 patients with focal AT (69 % vs. 100 % (n = 14) in ConvO, p = 0.01) and in 4 of 12 patients with a macro-reentry AT (31 % vs. 86 % (n = 12) in ConvO, p = 0.04). The termination rate of localized-reentrant ATs was 1 in 5 patients in the LM group only with the use of the algorithm (20 % vs. 100 % (n = 4) in ConvO, p = 0.05). The LM-algorithm was not superior for any type of AT. Furthermore, there was no statistical difference between the termination rates of the different ATs in the LM group (macro-reentry vs. focal AT: p = 0.25, focal vs. localized-reentry AT: p = 0.3, macro-reentry vs. localized-reentry AT: p = 1.0, overall comparison: p = 0.27).

### 3.4 Clinical outcome during follow-up

**Fig. 4:**
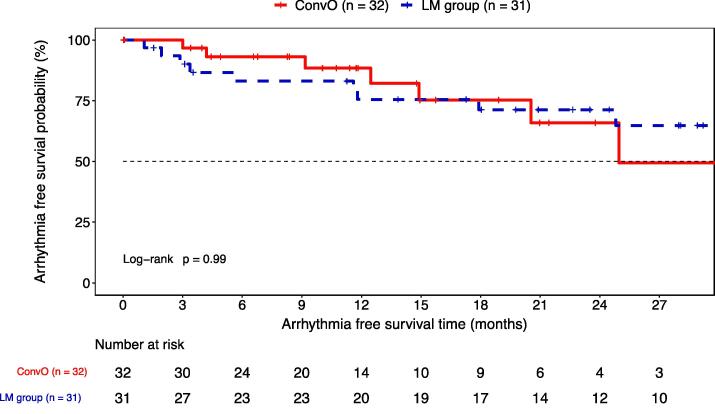
Kaplan-Meier curve of arrhythmia free survival. The red curve indicates the conventional only (ConvO) group. The blue curve represents patients of the LM group. There was no difference in overall outcome between the two groups in log-rank-test (p = 0.99) with a mean follow-up time of 20 ± 9 months. (For interpretation of the references to colour in this figure legend, the reader is referred to the web version of this article.)

During a mean follow-up of 20 ± 9 months, there were no differences in arrhythmia free survival between the two groups (Intention-to-treat analysis: LM group: 62 % vs. ConvO: 75 %, p = 0.3). 3 patients were lost to follow-up. The Kaplan-Meier arrhythmia-free survival analysis between the two groups is demonstrated in Fig. 4. The time to first recurrence, excluding events in the blanking period, was 12 ± 8 months with no differences between the two groups (13 ± 9 vs. 12 ± 8 months; p = 0.94).

### 3.5 Follow-up procedures

There was no difference in number of follow-up procedures (0.55 ± 0.89 LM group vs. 0.38 ± 0.61 ConvO, p = 0.7) and electrical cardioversions in the follow-up time (0.68 ± 1.7 vs. 0.5 ± 0.76, p = 0.5). Overall, 12 patients had one and 7 patients had two follow-up procedures. Of them, 14 patients suffered from atrial tachycardia and 5 from atrial fibrillation recurrence.

## 4 Discussion

### 4.1 Main findings of the study

This prospective, randomized study revealed the following main findings: (1) Conventional electrophysiological mapping was superior to electroanatomical mapping to identify AT mechanism and origin. (2) The overall procedure and fluoroscopy time was significantly longer in the LM group as compared to the conventional group. However, in patients with an appropriate identification of the AT mechanism, the procedural time to AT termination was not different. And finally, no difference was observed in terms of long-term freedom from arrhythmia after ablation between the two groups.

### 4.2 Ablation of atrial tachycardia using electroanatomic mapping systems

In this study, a conclusive LM could be created in 55 % of the cases, resulting in AT termination by ablation in 45 % based on LM algorithm information only. In 15 patients (45 %), the created map did not provide meaningful information regarding a specific AT mechanism and, therefore, the LM could not be used to define a specific ablation target. This observation had the following reasons: the LM tagged multiple sites as earliest activation, a significant map shifting occurred during or after mapping or only a few points could be recorded by the system due to wide areas of low-voltage zones, inaccurate annotation of early and late activation patterns and mismatches of window-of-interest settings. We used a decapolar lasso catheter as a standard mapping tool. The number of acquired points is therefore much lower as compared to the latest high-density catheters, which could have influenced the rate of correct AT mechanism identification [9].

The two critical steps in LM utilization are a thoroughly map creation and accurate interpretation, which in turn depends on correct settings on the one hand, but also on the fundamental technical limitations of the system on the other hand. Nevertheless, the presentation of the activation patterns by the system and the experience of the operator with both, the EAM system and AT mechanisms are further factors that may impact misinterpretations that therefore potentially result in high interobserver discrepancy [10].

### 4.3 Procedure duration and fluoroscopy using automated algorithm

Previous studies demonstrated a reduction of the radiation exposure to the patient with the application of 3-D electroanatomic maps [2], [3]. However, the overall procedure time and acute success rate was not different as compared to ablation procedures guided by conventional electrophysiological techniques [3]. Our study, the total procedure time was shorter, and the radiation exposure was lower in the conventional group as compared to the 3-D mapping group. Moreover, the efficacy of the 3-D mapping system to identify the critical site of the AT mechanisms was lower as compared to conventional mapping. These results may be explained by the following reasons: first, inaccurate or inappropriate local activity annotation may result in repeated mappings with reexamination of a specific location within the atrium or even re-mapping of the entire chamber. In our study the number of ATs was higher in the LM group, which could be caused by mislocalization and ablation at the wrong site with consequent altered AT circuit.

Second, the creation of a latency map requires more accuracy and therefore time than an anatomical 3-D map, since the correct parameter setting, the ConvO of the acquired points and the interpretation of the mapping are time-consuming. Romero and co-workers analyzed the procedural data of conventional- versus 3-D mapping-guided ablation performed by electrophysiological trainees [4]. Interestingly, their study yielded similar results: while the acute success rate was not different, a shorter procedure and fluoroscopy duration was observed in the conventional mapping group.

### 4.4 Algorithms for automated AT identification

Besides the algorithm used in our study, several other algorithms for AT mechanism identification exist, some of which have only recently been introduced. In the attempt to reduce the further above mentioned limitations, novel algorithms and 3-D propagation visualizations have been developed, such as ripple and COHERENT mapping of the CARTO system (Biosense Webster, USA) [11]. The ripple map represents the absolute value of the local time course of the voltage as a three-dimensional bar that changes in size and is therefore independent of the window-of-interest [12], [13], [14]. Moreover, the most recently introduced COHERENT mapping algorithm (CARTO, Biosense Webster), a graphical representation of a vector field that assigns a velocity vector to each point on the surface of a reconstructed cardiac anatomy, provides another new propagation visualization to identify AT mechanisms [15], [16]. Furthermore, ultra-high density mapping of complex AT with the RHYTHMIA system has been demonstrated to effectively identify the critical sites of the underlying arrhythmias without the need for additional conventional mapping [17]. Nevertheless, even with an increased sufficiency of 3-D activation mapping tools, conventional electrophysiological mapping techniques still have an important role in AT mapping, particularly to differentiate passive from active macro-reentrant circuits [18].

### 4.5 Study limitations

This small exploratory trial conducted at a single center was not powered and not designed to assess superiority of the LAT algorithm over conventional methods. The aim of the study was to gain experience of the reliability of the algorithm and to generate different hypothesis in which situations the algorithm could help in AT ablation.

The study was performed without the use of a high-density catheter; thus, the results may not be directly transferrable to AT ablations using this technology. Due to the limited number of patients, subgroup analysis was not feasible.

## 5 Conclusions

This small prospective, randomized study explored the performance of the automated NavX Ensite Precision latency-map (LM) algorithm. The use of the algorithm alone resulted in AT termination in 45 % of the patients, compared to 94 % using conventional methods. Significant improvements of contemporary electroanatomical mapping algorithms and high-density mapping techniques may still have the potential to improve outcomes. Larger studies are needed to assess the usefulness of automated AT mapping.

Funding: (None, no grant support).

Disclosures: (None).

Authors Contributions: Spittler: manuscript and statistical analysis; Witte: Data acquisition and follow-up; Hoffmann, Konrad and Rostock performed the procedures; Marx, Mollnau and Quesada-Ocete: patient inclusion. All revised the manuscript and the results.

Registration: This study was not registered.

## Declaration of Competing Interest

The authors declare that they have no known competing financial interests or personal relationships that could have appeared to influence the work reported in this paper.
